# Deciphering the PgLEA2-50 interactome: implications for abiotic stress responses in ***Panax ginseng***

**DOI:** 10.1080/15592324.2026.2624961

**Published:** 2026-02-05

**Authors:** Qi Wang, Jinlong Liu, Mengyang Zhang, Peiying Wang, Tong Li, Xingbo Bian, Xiaoyun Chen, Shuang Chen, Lina Wang, Juntao Lei, Liu Han, Mengran Xu, Qiuyue Zhang, Xiujuan Lei, Yingping Wang, Xin Sun

**Affiliations:** a College of Pharmacy, Jilin Medical University, Jilin, China; b Outpatient Department, College of Chinese Medicinal Materials, Jilin Agricultural University, Changchun, China; c Changtong Community Health Service Center, Changchun, China; d Imaging Center, Jilin People's Hospital, Jilin, China

**Keywords:** LEA, abiotic stress, IP-MS, gibberellin-signaling, DELLA

## Abstract

Ginseng's prolonged development renders it susceptible to environmental stresses. Late embryogenesis abundant (LEA) proteins are essential for plant resistance to abiotic stress. Our previous study demonstrated that *PgLEA2-50*, a member of the LEA protein family, plays a significant role in stress resistance. In this study, we employed IP-MS, bioinformatics, and molecular interaction assays to investigate the mechanisms underlying its stress resistance. PgLEA2-50 formed complex networks with multiple interacting proteins, which were enriched in stress-related processes such as gibberellin (GA) signal transduction, saponin biosynthesis, and the oxidative stress response. Transcriptome analysis revealed that its interacting targets exhibited significant responses to abiotic stress at the transcriptional level. An investigation of the DELLA protein PgRGA4 showed that it was down-regulated following GA induction, with its transcriptional activity inhibited under stress conditions. *PgRGA4* was found to be localized in both the nucleus and cytoplasm, and co-immunoprecipitation (CO-IP) confirmed its interaction with PgLEA2-50, suggesting that PgLEA2-50 indirectly regulates GA-mediated stress resistance. This study provides a ginseng-specific case for the role of LEA proteins in stress resistance and identifies a novel gene target for molecular breeding in medicinal plants.

## Introduction

The growth and development of plants are significantly influenced by environmental factors. Although plants typically maintain a dynamic equilibrium with their surroundings, they are inevitably subjected to adverse effects from various environmental stresses.^1^ Among these, numerous hazards arise from abiotic factors, the causes of which are multifaceted. Furthermore, the inherent immobility of plants imposes survival challenges in specific environments. *Panax ginseng*, a perennial herb belonging to the *Araliaceae* family, is renowned for its considerable medicinal and economic value.^2^ As a quintessential geo-authentic medicinal material, ginseng has stringent requirements for its growth environment.^3^ Coupled with its lengthy growth and development cycle, this makes it particularly vulnerable to adverse influences such as water availability, temperature fluctuations, salinity, and metal exposure.^4^ The ability to adapt and resist stress is crucial for plants to endure challenges and ensure their growth, development, and reproduction. The extended evolutionary history of plants has endowed them with a repertoire of functional genes and stress resistance mechanisms that enable them to respond to and withstand abiotic stresses.^5^

The mechanism of cellular resistance to abiotic stress operates at multiple levels. In addition to regulating enzymatic reactions, molecular mechanisms, and gene expression, the protection of cellular composition and structure is also critical.^6^ In this context, LEA proteins are indispensable.^7^^,^^8^ These proteins exhibit a strong affinity for water and possess a high water-holding capacity, which helps prevent damage to cellular structures and the denaturation or inactivation of functional proteins, thereby enabling resistance to various types of abiotic stress.^9^ Under stress conditions that induce protein aggregation, misfolding, and even denaturation, the disordered and flexible structure of LEA proteins serves as a physical barrier between enzymes and protein molecules.^10^ By safeguarding these components, LEA proteins indirectly maintain the stability of physiological activities, and this protective mechanism is predicated on protein–protein interactions.^11^

Numerous studies have elucidated the mechanism of action of LEA proteins, suggesting that they function similarly to “molecular chaperones” by serving as external components that mitigate the damage caused by cellular dehydration to sensitive proteins.^12^ Despite these similarities, LEA proteins operate independently of ATP in their protective roles, indicating a fundamental difference between the mechanisms of LEA proteins and “molecular chaperones”.^13^ Current research on LEA proteins primarily concentrates on their inherent disordered structures and their protective roles for various cellular components. Thermophoresis analysis quantitatively confirmed the binding of the LEA protein to its target protein. Moreover, with the intensification of H_2_O_2_-mediated stress, the affinity between the LEA protein and its interacting protein was enhanced.^14^ Numerous experimental studies on plant LEA proteins have also confirmed the crucial role of these proteins in stress resistance. The LEA member *MtCAS31* of *Medicago truncatula* is predominantly expressed in nodules and interacts with leghemoglobin MtLb120-1. The protective function of MtCAS31 on the target protein MtLb120-1 prevents its denaturation and inactivation under heat stress.^15^ In similar studies, it was found that the dehydrin protein ERD14 of *Arabidopsis thaliana* interacts with both glutathione S-transferase (GST) and catalase (CAT). Researchers have speculated that, in addition to being protected by LEA proteins, the binding of these antioxidant enzymes to ERD14 may enhance their activity, thereby reducing oxidative stress generation.^16^ The *Pisum sativum* PsLEAm protein, located in the mitochondrial matrix, belongs to the LEA3 subfamily. *In vitro* drying experiments have demonstrated that the recombinant PsLEAm protein exerts protective effects on both fumarase and thiocyanate enzymes.^17^ Additionally, Shiraku et al. identified proteins interacting with GhLEA3 from a cotton yeast library. Among these, the target gene GhVDAC1 is associated with both the calcium signaling pathway and the cGMP-PKG signaling pathway, whereas GhGAPA functions in glycogen biosynthesis and metabolic pathways. Both genes are positively induced by drought stress, and their expression levels are significantly reduced in *GhLEA3*-knockout cotton lines.^18^ These studies demonstrate the broad specificity of LEA proteins in targeting various proteins, highlighting interspecies variability. Furthermore, they confirm the significant protective role of LEA proteins in enhancing plant resistance to stress.

In our recent study on the stress resistance of ginseng, we discovered that PgLEA2-50, a member of the LEA2 subfamily, significantly responds to drought and extreme temperature stress. Transgenic tobacco lines expressing PgLEA2-50 exhibited enhanced stress tolerance and improved physiological status.^19^ While PgLEA2-50 demonstrates remarkable stress resistance in both ginseng and model plant transformations, its regulatory mechanism remains unclear. The function of a specific protein is typically determined by its interactions with multiple proteins, forming a complex network.

To further investigate the mechanism of PgLEA2-50, this study employed immunoprecipitation-mass spectrometry (IP-MS) to identify proteins that interact with the target protein. Additionally, bioinformatics methods were utilized for a systematic analysis. Among the series of target proteins identified through screening, a subset was found to be involved in the regulation of GA-mediated signaling pathways. Consistent with our previous study, which confirmed that *PgLEA2-50* exhibits a significant transcriptional response to GA,^19^ this observation prompted us to focus our research on DELLA protein, which is the key negative regulators of the GA signaling pathway. The crucial interaction relationships were subsequently validated using a ginseng protoplast-mediated transient transformation system and co-immunoprecipitation (CO-IP). This study not only provides data support and a theoretical foundation for elucidating the mechanisms of action of key LEA genes in ginseng but also offers insights and methodologies for researching functional genes in ginseng.

## Results

### Transient expression of PgLEA2-50 in protoplasts and enrichment of positive cells

**Figure 1.**
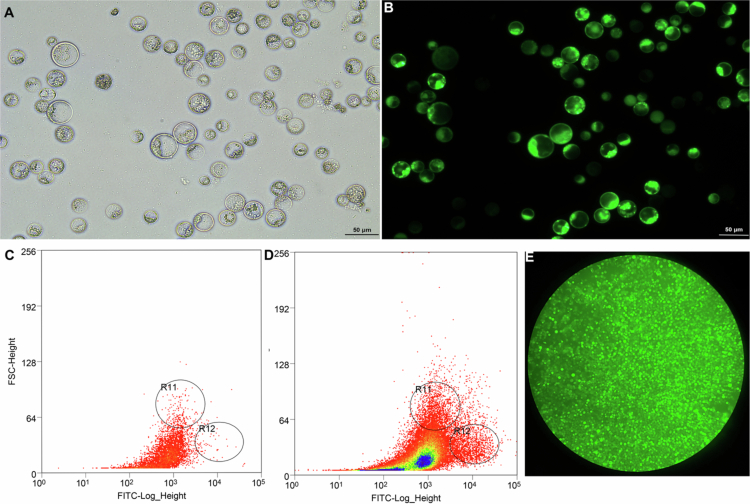
Transformation of *PgLEA2-50* in protoplasts and enrichment of positive cells by flow cytometry. (A) Ginseng embryoid protoplasts under bright field. (B) Viability detection of protoplasts under 488 nm excitation was based on FDA staining. (C) Scatter plot of control protoplast sample analyzed by flow cytometry. (D) Scatter plot of transfected PgLEA2-50-GFP protoplast samples analyzed by flow cytometry. (E) Microscopic observation of the enrichment effect of positive cells under a 5× objective lens.

Microscopic enumeration using a hemocytometer revealed that the protoplast yield exceeded 1 × 10^7^ cells/mL (Figure 1A), and FDA staining confirmed that over 90% of the protoplasts were viable (Figure 1B). Under 488 nm laser excitation, fluorescence microscopy visualized the successful expression of the PgLEA2-50-GFP fusion protein. Flow cytometric analysis further demonstrated a specific cell population with significantly enhanced fluorescence intensity in the transfected cell population compared to the empty vector GFP control group (Figure 1C and D). By gating this fluorescent population and applying screening thresholds based on forward scatter (FSC) and side scatter (SSC) parameters, we applied the fluorescence signature to whole-sample event analysis, enabling the precise sorting of positive cells. After enrichment by flow cytometry based on fluorescent signal intensity, a large number of positive cells in the sample were screened, and the enrichment effect could be observed under a microscope (Figure 1E).

### Silver staining validation of positive protoplasts in IP assays

**Figure 2.**
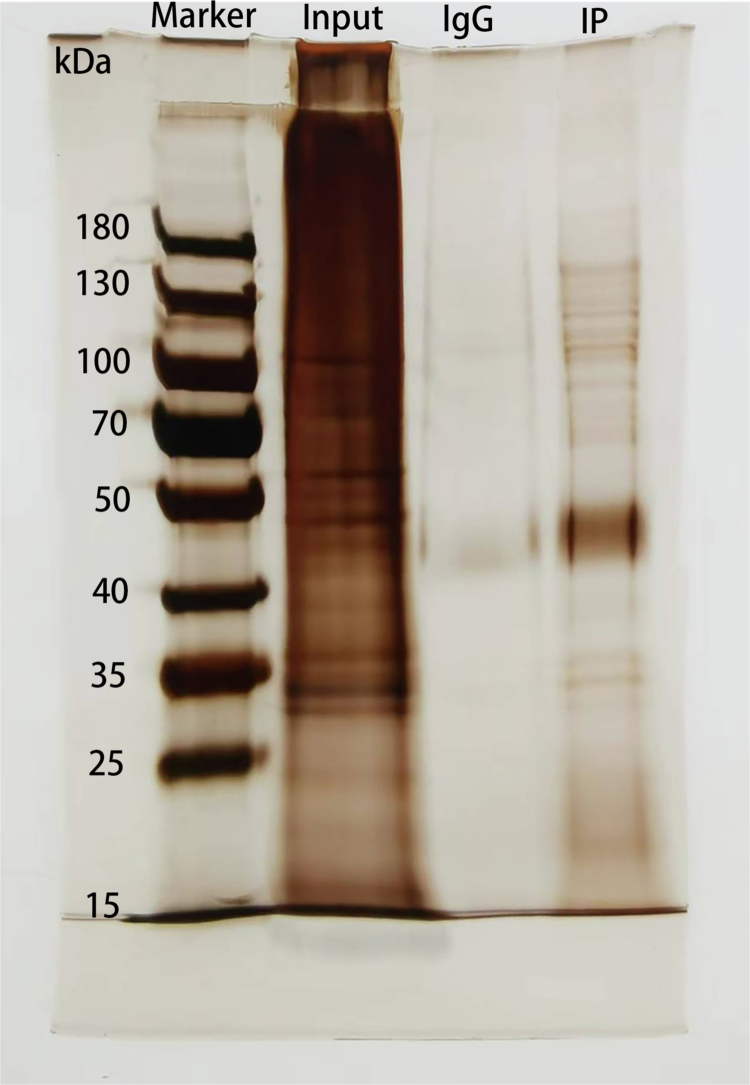
Silver staining detection results of the control group and the experimental group.

The total protein from ginseng protoplasts in both the control and positive transformation groups was extracted, and a standard curve was generated at concentrations of 0, 0.025, 0.05, 0.1, 0.2, 0.3, 0.4, and 0.5 mg/mL. The *R*² value of the fitting curve was greater than 0.995, which aligns with the standard for determining protein concentration (Figure S1). Following this determination, the prepared protein sample met the criteria for immunoprecipitation. The results from silver staining indicated that the input samples exhibited good integrity and abundance, with minimal degradation observed. Fewer bands were detected in the IgG group, suggesting that it may bind to only a small number of non-specific proteins. In contrast, a greater number of interacting proteins with the target protein were identified in the IP group, demonstrating that the protein solution sample was suitable for mass spectrometry detection and analysis (Figure 2).

### Identification of PgLEA2-50 interacting proteins by mass spectrometry

**Figure 3.**
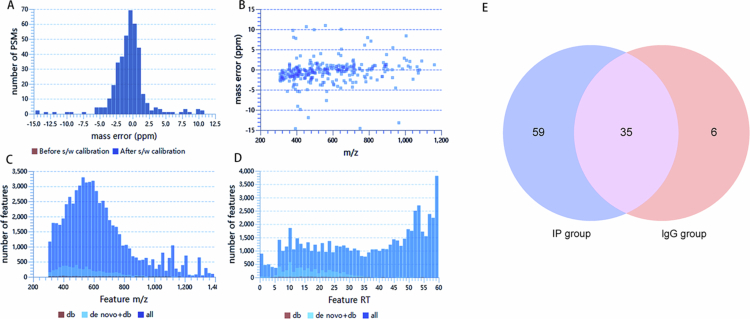
Mass spectrometry characteristics and protein Venn diagram. (A and B) were the mass precursor errors of peptide mass matching (PSM) in the filtering results. (C) The detectable feature in the m/z range. (D) The number of detectable features in each RT interval. (E). Venn diagram of proteins detected in the IP group and IgG group.

The quality control results regarding experimental accuracy and the detection of peptide feature distribution indicated that the experiment was both highly accurate and reliable (Figure 3A–D). As illustrated in the Venn diagram depicting the proteins identified in the IP and immunoglobulin G (IgG) groups, a total of 94 proteins were identified in the IP group, while 41 proteins were found to be non-specifically bound by IgG, 35 of which were also retrieved in the IP group (Figure 3E). The proteins within this intersection were primarily ribosomal proteins and their subunits, as well as proteins related to photosynthesis, including glyceraldehyde-3-phosphate dehydrogenase and ATP synthase subunits. After excluding non-specific proteins from the control group, 59 proteins potentially interacting with PgLEA2-50 were detected in the IP group.

Further protein sequence alignment revealed duplications and high homology among these genes. These sequences were then subjected to BLAST analysis against the ginseng protein library to match the IDs of these proteins within the ginseng genome for subsequent analysis. Following the removal of redundant sequences, 50 interacting proteins remained, and the information of the interacting proteins is presented in Table 1. Then the conserved domains of the identified genes were analyzed using the SMART database (http://smart.embl.de/), leading to the exclusion of genes with missing or incomplete target domains. The results demonstrated that each of the identified genes harbored intact conserved domains (Figure S2). The results indicated that a diverse array of proteins interacted with PgLEA2-50, including stress-resistant proteins, transporters, oxidoreductases, antioxidant enzymes, and proteins involved in saponin synthesis. Additionally, several members of the same gene family were identified, such as cytochrome P450, glycosyltransferase, and multidrug resistance proteins (Table 1).

**Table 1.** Mass spectrometry analysis and database identification of PgLEA2-50 interacting proteins.

Uniprot number	Genome ID	Gene description information
A0A514EJU0	EVM0013493	Non-specific serine/threonine protein kinase
C7DZJ5	EVM0064045	Spermidine synthase
A0A8T9ICV9	EVM0019265	SLEEPY1 and SLY1-1
B5THI3	EVM0041938	Major latex-like protein
A0A060IMN8	EVM0020372	Actin 1
A0A0Y0ASU5	EVM0029980	Actin-11
Q9MAV9	EVM0000557	Cytoplasmic ribosomal protein S13
O82146	EVM0062132	Beta-amyrin synthase 2
A9Z0Q0	EVM0030395	Catalase
A0A8T9ICW4	EVM0019202	DELLA protein and PgRGA4
D3JX88	EVM0064248	Glutamate decarboxylase
B2MVQ2	EVM0042899	Glutathione S-transferase
A0A220QKC7	EVM0027231	Ascorbate peroxidase
A0A220QKD0	EVM0012365	Ascorbate peroxidase
A0A290G3G2	EVM0027231	Ascorbate peroxidase
W8CR43	EVM0007753	accD
Q68RZ7	EVM0007753	accD
A0A060ID18	EVM0024396	ADP-ribosylation factor
Q20BN2	EVM0008663	ADP-ribosylation factor
A0A385CI77	EVM0021074	Alkyl transferase
Q75W19	EVM0045786	Cytochrome P450
H2DH21	EVM0047897	Cytochrome P450
H2DH18	EVM0040226	Cytochrome P450
H2DH24	EVM0017877	Cytochrome P450
H2DH20	EVM0004859	Cytochrome P450 CYP71D313
A0A3Q9BHB7	EVM0065722	Cytochrome P450 CYP74B32
A0A060IGR7	EVM0018510	Elongation factor 1-alpha
A0A0Y0AU34	EVM0014750	Elongation factor 1-gamma and EF1-gamma
A0A7L9CQR2	EVM0042455	Translation elongation factor 1-alpha
A0A0D5ZDI3	EVM0056072	Glycosyltransferase
A0A0D5ZCT2	EVM0035444	Glycosyltransferase
A0A0D5ZDG6	EVM0041989	Glycosyltransferase
A0A7G3KHF5	EVM0050262	Glycosyltransferase
A0A0D5ZD66	EVM0063037	Glycosyltransferase and UGTPg33
A0A0D5ZD60	EVM0050262	Glycosyltransferase and UGTPg43
A0A288SSS7	EVM0036782	MYB transcription factor protein 7 and PgMYB7
A0A7L4V011	EVM0027908	Lipoxygenase
Q68RV2	EVM0008078	NAD(P)H-quinone oxidoreductase subunit I
W8CQY3	EVM0008078	NAD(P)H-quinone oxidoreductase subunit I
Q68S02	EVM0050926	NAD(P)H-quinone oxidoreductase subunit K
W8CPY2	EVM0050926	NAD(P)H-quinone oxidoreductase subunit K
V9PEW5	EVM0028130	Phosphomevalonate kinase
F2ZBW0	EVM0022738	PgMADS protein3
A0A1P7Y0D9	EVM0014081	Pleiotropic drug resistance transporter
A0A1P7Y0D6	EVM0009267	Pleiotropic drug resistance transporter
A0A1P7Y0D8	EVM0009267	Pleiotropic drug resistance transporter
A0A1P7Y0D5	EVM0044779	Pleiotropic drug resistance transporter
A0A096VPM7	EVM0028199	Pleiotropic drug resistance transporter 1
A0A096VPN4	EVM0044779	Pleiotropic drug resistance transporter 2
U5KP94	EVM0003995	Pleiotropic drug resistance transporter 3
A0A060IGT8	EVM0057963	Putative polyubiquitin
B8YDG5	EVM0004384	Short-chain alcohol dehydrogenase
A0A8K1HT57	EVM0012140	Terpene cyclase/mutase family member
Q307T0	EVM0055859	Tonoplast intrinsic protein
A0A060ID32	EVM0001849	Tubulin alpha chain
A0A060IGE0	EVM0007078	Tubulin beta chain
A0A0K0PVL3	EVM0046042	UDP-glucosyltransferase 103
A0A0A6ZFY4	EVM0050262	UDP-glucosyltransferase 29
A0A0K0PVW1	EVM0046042	UDP-glycosyltransferase

### Functional enrichment analysis of PgLEA2-50 interactome via GO databases

**Figure 4.**
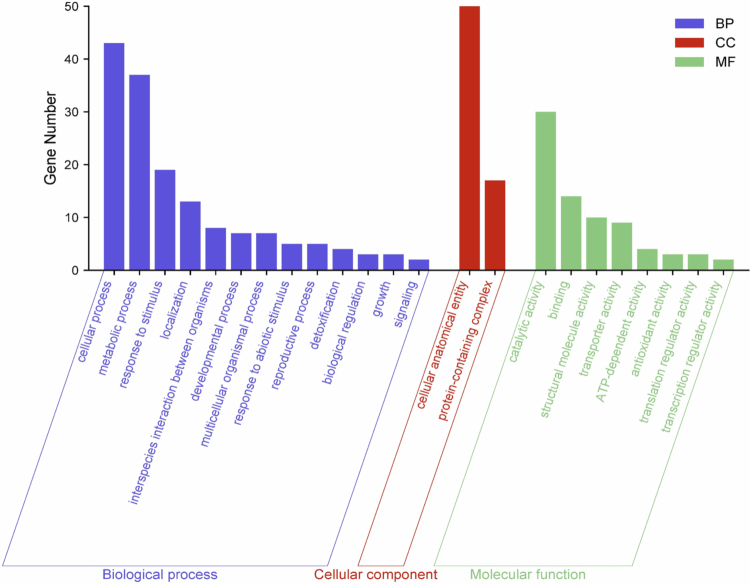
GO enrichment analysis of PgLEA2-50-interacting proteins.

The GO and KEGG analyses of the target genes of PgLEA2-50 effectively elucidated the biological functions and processes in which these genes were typically involved. During the GO analysis, the interacting proteins were found to be enriched in various pathways related to biological processes, cellular components, and molecular functions (Figure 4). These proteins were significantly involved in ginseng reproduction (GO: 0000003), growth (GO: 0040007), and development (GO: 0040007), as well as participating in signal transduction (GO: 0023052), biological regulation (GO: 0044419), and metabolism (GO: 0008152). They also responded to both biological and non-biological stimuli (GO: 0044419, GO: 0022414). In terms of cellular components, the target proteins were primarily concentrated in the cell anatomical entity (GO: 0110165) and protein complexes (GO: 0032991). The molecular function analysis indicated that many target proteins possess specific catalytic abilities (GO: 0003824) and the capacity to bind to other components (GO: 0005488). Additionally, some proteins exhibited regulatory activities related to transcription (GO: 0140110) and translation (GO: 0045182). Furthermore, three target proteins were identified to have antioxidant activity (GO: 0016209).

### Functional enrichment analysis of PgLEA2-50 interactome via KEGG databases

**Figure 5.**
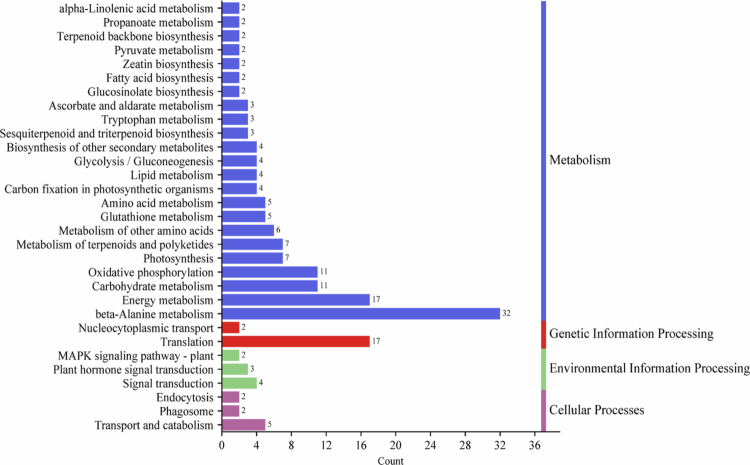
KEGG enrichment analysis of PgLEA2-50-interacting proteins.

To analyze the KEGG pathway, 50 target proteins of PgLEA2-50 were annotated within the established threshold, resulting in their classification into 31 KEGG metabolic pathways (Figure 5). These pathways encompass metabolism, genetic information processing, environmental information processing, and cellular processes. The interacting proteins of PgLEA2-50 were enriched predominantly in energy metabolism, oxidative phosphorylation, glycolysis or gluconeogenesis, and photosynthetic processes. Furthermore, they play a crucial role in the biosynthesis of various organic compounds, including terpenoids, amino acids, lipids, and zeatin. Environmental information processing primarily involves plant hormone signal transduction and the MAPK signaling pathway. Additionally, pathways related to nuclear transport and translation, as well as those involved in transport and catabolism, phagosome, and endocytosis within cellular processes, were also enriched.

### Transcriptomic analysis of protein–coding genes interacting with PgLEA2-50

**Figure 6.**
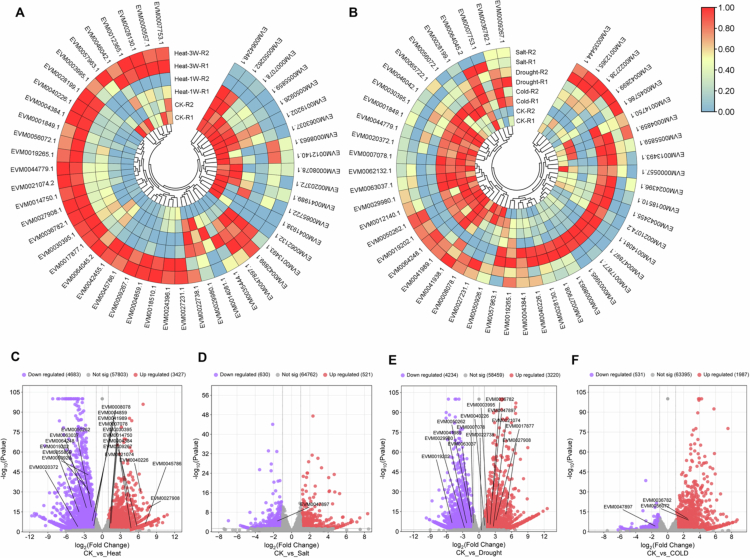
Transcriptome analysis and differential gene screening of PgLEA2-50 target protein-coding genes under abiotic stress. (A) Heatmap of target genes expression under high-temperature stress. (B) Heatmap of target genes expression under salt, drought and cold stress. (C) Volcano plot of the differential expression of target genes under high-temperature stress. (D) Volcano plot of the differential expression of target genes under salt stress. (E) Volcano plot of the differential expression of target genes under drought stress. (F) Volcano plot of the differential expression of target genes under cold stress.

The heat map of gene expression indicated that the transcription levels of members interacting with PgLEA2-50 were significantly induced by abiotic stress, with variations in both the type and degree of response (Figure 6A and B). Under high-temperature stress, a total of 19 genes were differentially expressed, including nine that were up-regulated and ten that were down-regulated (Figure 6C). Under drought stress, seven genes were up-regulated while seven were down-regulated (Figure 6D). Additionally, two genes were up-regulated and one was down-regulated under low-temperature stress (Figure 6E). Only one gene exhibited a down-regulated response to salt stress (Figure 6F).

### Cloning and expression analysis of PgRGA4 in response to GA treatment

Among the target proteins, *PgRGA4* belongs to the DELLA family, a class of core negative regulators in the GA-signaling pathway. Transcriptomic data analysis demonstrated that PgRGA4 was responsive to abiotic stresses and showed a downregulated expression pattern under GA treatment. In contrast, the transcriptional level of *PgLEA2-50* was significantly upregulated upon GA induction. This gene appears to be associated with PgLEA2-50 in the GA pathway, and this research implication was the key rationale for selecting it as the subject of subsequent investigations.

**Figure 7.**
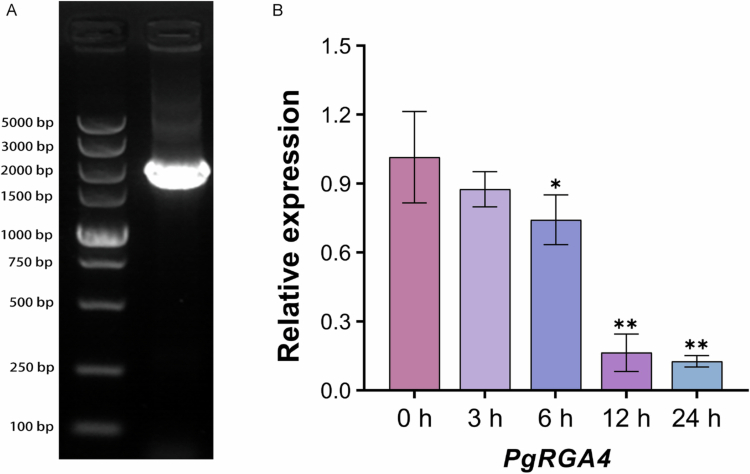
Cloning of *PgRGA4* and analysis of expression patterns at different time points under GA treatment. (A) Electrophoretogram of target gene cloning. (B) The qRT-PCR detection of *PgRGA4* under GA treatment (*p < 0.05, **p < 0.01, and the error bars represented the SD values of replicates).

The full-length coding sequence of the *PgRGA4* gene is 1758 bp, consisting of 585 amino acids, with a molecular weight of approximately 64.19 kDa. The results of gel electrophoresis for the cloned *PgRGA4* gene demonstrated that the band size was consistent with expectations (Figure 7A). Further gene sequencing and alignment confirmed that the cloned sequence was entirely consistent with the genomic data (Table S1). The qRT-PCR analysis of *PgRGA4* under exogenous GA application demonstrated a gradual decrease in the transcription level with the extension of GA induction time (Figure 7B). This finding suggested that GA inhibits the transcription of *PgRGA4* in ginseng, indicating a potential negative regulatory role.

### Subcellular localization of PgRGA4

**Figure 8.**
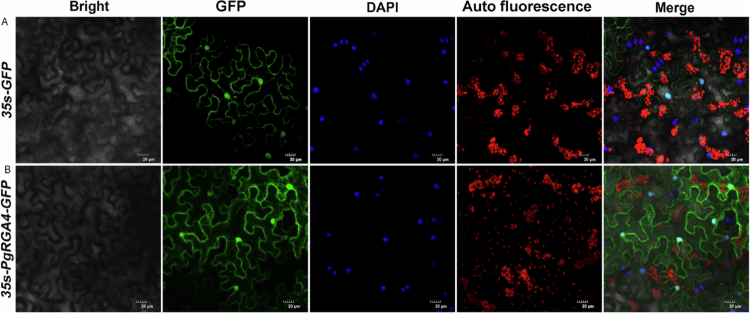
Subcellular localization of *PgRGA4-GFP* fusion protein. (A) Expression of the empty *35s-GFP* vector without the target gene in tobacco leaves. (B) Expression of *35s-PgRGA4-GFP* fusion protein in tobacco leaves. Images were presented in the following sequence from left to right namely bright field, localization of GFP or the fusion protein, detection of chloroplast autofluorescence, DAPI staining for nucleus, and merged images.

To determine the subcellular localization of *PgRGA4*, two constructs were analyzed, including the empty vector *35s-GFP* and the fusion construct *35s-PgRGA4-GFP* (Figure 8). Notably, chloroplast autofluorescence was employed to eliminate non-specific background interference, thereby ensuring the accuracy of the localization results. As shown in the empty vector control, the free GFP protein exhibited a strong fluorescence signal in the nucleus, which completely overlapped with partial DAPI-stained nuclear regions, while displaying uniformly diffuse signals in the cytoplasm (Figure 8A). Combined with the validation from the control group and autofluorescence channel, *PgRGA4* was confirmed to be specifically localized in the nucleus and cytoplasm (Figure 8B).

### CO-IP verification of the interaction between PgRGA4 and PgLEA2-50

**Figure 9.**
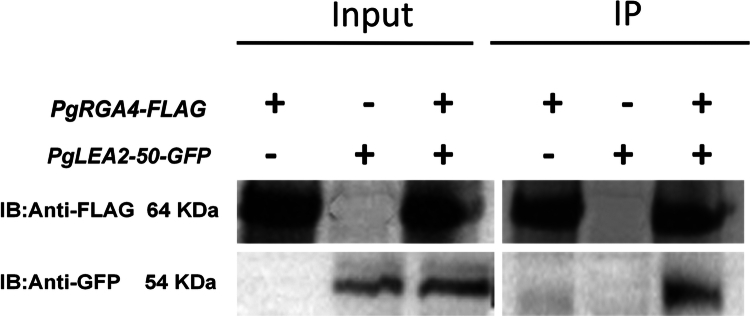
CO-IP verification of the interaction between PgLEA2-50 and PgRGA4 protein. “+” was the overexpression of the corresponding target gene in the protoplast sample, and “−” was the non-overexpression of the gene. The expected size of PgLEA2-50-GFP fusion protein was about 54 kDa, and the expected size of PgRGA4-FLAG fusion protein was about 64 kDa.

**Figure 10.**
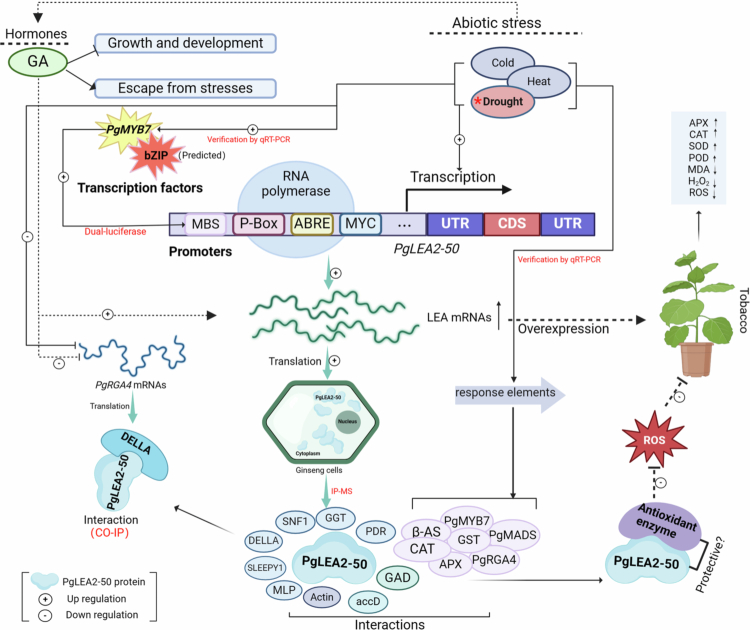
A hypothetical abiotic stress response model of PgLEA2-50 based on interaction and transcriptomic data.

The seamless cloning process, utilizing *BamHI* as the restriction site, successfully ligated the target gene fragment to the vector backbone, resulting in a sequence consistency of 100% (Figure S3). To exclude the interference of false positive results, protoplast samples transformed with PgLEA2-50-GFP or PgRGA4-FLAG alone were established. The results indicated that the target fusion protein could be detected with specific antibodies in the input group, and the band position was consistent with the expected size (Figures 9 and 10). In the protoplasts co-transformed with the two plasmids, both the tagged proteins GFP and FLAG antibodies detected the target proteins in the sample. In the IP group, the samples were immunoprecipitated using magnetic beads containing FLAG antibodies. After Western blot (WB) verification, the samples transformed with only GFP did not show bands, while the samples transformed with only FLAG displayed target bands post-WB verification. Furthermore, both GFP and FLAG labels were detected in the co-transformed samples, indicating that the PgRGA4-FLAG protein was pulled down by FLAG magnetic beads in conjunction with PgLEA2-50-GFP, further confirming the interaction between these two proteins. In addition, the original images of the experiments in this chapter have been uploaded to the supplementary files.

## Discussion

The response of plants to abiotic stress and the enhancement of stress resistance are dynamic and complex processes influenced by the interplay of multiple genes and signaling pathways.^20^ Systematic research on key gene interaction proteins can overcome the limitations of single gene function analysis and elucidate the core nodes of stress resistance regulation from a network perspective. Utilizing the principle of immunoprecipitation, IP-MS technology captures target proteins and their interacting partners through specific antibodies and employs mass spectrometry to analyze protein components.^21^ This analysis not only elucidates the interaction relationships but also provides theoretical support for constructing protein interaction networks. In this study, the fusion protein of PgLEA2-50 and GFP was employed to enrich interacting protein members from the total protein of protoplasts using a ginseng transient expression system. Compared to other interaction research methods, the proteins expressed in this system undergo natural processing, modification, and maturation, thereby reflecting protein characteristics more accurately under physiological conditions. Consequently, the identification of interaction relationships exhibits higher accuracy and sensitivity, free from interference by self-activation effects. Since no independent mass spectrometry samples transfected with GFP alone were set up, there may be a risk of false positives among the target proteins identified by IP-MS, such as non-specific binding to the GFP protein. This is an unavoidable limitation arising from the experimental design. Therefore, CO-IP validation is essential before conducting an in-depth investigation into the protein‒protein interactions.

From a bioinformatics perspective, 50 proteins that bind to PgLEA2-50 were enriched in various biological processes and metabolic pathways, collaborating with PgLEA2-50 to withstand extreme environments through complex cascades and synergies. The interaction between PgLEA2-50 and its diverse target proteins may represent an important prerequisite for the manifestation of its stress resistance function, suggesting a potential link between these interaction characteristics and stress tolerance phenotypes.

It is noteworthy that numerous potential interacting proteins were closely associated with plant responses to abiotic stress in our research. Among these, spermidine serves a dual role by directly protecting cellular components and modulating stress signaling pathways. Additionally, spermidine synthase exhibits multiple regulatory functions in the plant stress response by efficiently catalyzing the propylamine transfer reaction during spermidine biosynthesis. The experimental data indicate that the transcriptional level of ginseng spermidine synthase is significantly up-regulated following treatments with ABA, MeJA, mannitol, and heavy metals. Notably, under cold and salt stress conditions, the increase in its expression is particularly pronounced.^22^ The PDR subfamily of ABC transporters in plants is capable of transporting metal ions, plant hormones, and terpenoids, and it responds widely to abiotic stresses indirectly.^23^ Currently, the PDR gene family in ginseng has been identified and confirmed to play a role in the transport of saponins and in the response to abiotic stress.^24^ In this study, IP-MS screening combined with verification confirmed that PgLEA2-50 can form specific interactions with multiple members of the PDR gene family. Moreover, PgLEA2-50 may interact with E3 ubiquitin ligase, glycosyltransferase, quinone oxidoreductase, and others. From the perspective of LEA mechanisms, the binding of LEA to interacting proteins primarily serves to protect the structure and activity of the target protein. However, the targets vary between different species and different LEA proteins. Furthermore, interactions between LEA and actin, as well as antioxidant enzymes, have been reported in studies examining LEA-interacting proteins in other species.^9^^,^^25^^,^^26^

DELLA protein serves as a key negative regulator in the gibberellin signaling pathway.^27^ It inhibits the expression of genes associated with gibberellin-mediated stress resistance, thereby delaying plant growth.^28^ The mechanism helps maintain the negative regulation of gibberellin pathway genes and slows down plant growth and development, enabling organisms to allocate more energy and metabolites to resist stressful environments.^29^ Such negative feedback regulation may be crucial for maintaining a balance between growth and defense in plants. The stability of DELLA protein is regulated by the level of GA. When the concentration of GA reaches a specific threshold, it binds to the GID1 receptor, forming a complex that targets the DELLA protein.^30^ This complex, which is mediated by a ubiquitin ligase, leads to the ubiquitination and subsequent degradation of the DELLA protein. In this experiment, the qRT-PCR analysis of the *PgRGA4* coding gene in response to exogenous GA application indicated that GA may inhibit the synthesis of DELLA protein at the transcriptional level. Transcriptome data revealed a significant decrease in the transcription level of *PgRGA4* under high-temperature and drought conditions. The interaction between PgLEA2-50 and PgRGA4 is hypothesized to potentially exert a protective effect on PgRGA4 structure and activity, which could reduce protein degradation and damage under stress conditions. Combined with previous findings showing that *PgLEA2-50* is predominantly expressed in the cytoplasm, we speculate that PgLEA2-50 and PgRGA4 may have potential functional crosstalk in the cytoplasmic compartment.^19^ Although CO-IP experiments have confirmed the interaction between PgLEA2-50 and DELLA, further analysis of this relationship, as well as a deeper understanding of the underlying mechanisms, necessitates more detailed research. Based on current research findings, we propose the following hypothesis: under stress conditions, PgLEA2-50 may maintain PgRGA4 protein stability through specific interaction, thereby indirectly influencing the regulatory balance of the GA signaling pathway and supporting plants in optimizing energy and resource allocation. This hypothesis provides a direction for future in-depth exploration of their functional associations.

Under stress conditions such as drought and high temperatures, the secondary metabolites in plants increase to enhance their resistance to stress.^30^ The pharmacological value of ginseng primarily stems from ginsenosides. Studies have demonstrated that the content of ginsenosides increases under specific adverse conditions. Moreover, moderate and timely abiotic stress exerts a positive impact on enhancing the medicinal activity of ginseng and promoting the formation of its value.^31^ This outcome is closely linked to the enhancement of enzyme activity associated with saponin synthesis and metabolism. This study demonstrated that PgLEA2-50 interacts with several enzymes involved in the regulation of saponins. Notably, the target protein β-AS (O82146) plays a crucial role in the biosynthetic pathway of ginseng oleanane triterpenoid saponins. Additionally, the target proteins UGTPg100 (A0A0K0PVW1) and UGTPg1 exhibit high homology and are capable of specifically catalyzing the C6-OH position of glycosylated protopanaxatriol (PPT) to produce the biologically active ginsenoside Rh1.^32^ Based on the above interaction results, we speculate that PgLEA2-50 may indirectly support ginsenoside biosynthesis during abiotic stress by protecting the structure and activity of these key saponin synthesis enzymes. The potential regulatory pathway could enhance the activity of secondary metabolic pathways, thereby promoting ginsenoside synthesis. This finding is anticipated to contribute to a deeper understanding of the internal mechanisms underlying the increased accumulation of secondary metabolites, such as saponins, in ginseng under abiotic stress.

The protective role of LEA proteins against oxidative stress and the preservation of functional protein activity and structure is a crucial mechanism for enhancing plant stress resistance. In this study, enzymes such as CAT, ascorbate peroxidase (APX), and GST, which possess reactive oxygen species (ROS) scavenging activity, were identified as functional proteins that significantly contribute to abiotic stress responses. IP-MS screening suggested that these enzymes may potentially interact with PgLEA2-50. We speculate that PgLEA2-50 may exert a potential protective effect through physical binding to these ROS-scavenging enzymes, which could represent an important potential mechanism underlying the formation of ginseng stress resistance and the enhanced stress tolerance of the transgenic lines.

Based on the experimentally verified findings of this study, transcriptomic differential expression patterns, and prior bioinformatics analyses, we put forward a hypothetical molecular mechanism underlying the role of PgLEA2-50 in plant abiotic stress resistance. In future studies, we will further validate the key regulatory relationships in the aforementioned model, investigate the specific roles of PgLEA2-50 in the GA signaling pathway and saponin synthesis under stress conditions, and conduct a more in-depth analysis of the functional mechanisms of key genes in ginseng.

## Materials and methods

### Transient overexpression of PgLEA2-50 in ginseng protoplasts

Ginseng embryoids were dissociated in a medium containing 2% (w/v) cellulase R-10 (Yuanye, S10042), 0.5% (w/v) pectinase Y-23 (Yuanye, S10008), and 0.7 M mannitol, in accordance with our previous research findings^33^ The protoplasts were subsequently purified at 800 rpm for approximately 7 h, with viability assessed using fluorescein diacetate (FDA). The *PgLEA2-50* expression plasmid was extracted in large-scale and quantified to a concentration of 1000 ng/μL. A total of 10 μg of plasmid was used per 100 μL of purified protoplasts and was transformed using 40% polyethylene glycol (PEG)-4000 for a duration of 15 min. Following transformation, 440 μL of W5 solution was added to halt the reaction, and the supernatant was discarded after centrifugation at 1000 r/min for 5 min. Finally, 200 μL of MMG solution was added for resuspension, and the protoplasts were cultured in the dark for 16–24 h.

### Flow cytometry enrichment of positive cells and total protein extraction

The Flow-check Pro Fluorospheres were utilized to calibrate the optical path of the flow cytometer (Beckman, MoFlo XDP). The excitation light employed was 488 nm blue light, and the 530/30BP-FITC channel served as the receiving channel to detect the green fluorescence signal emitted by the GFP protein. The voltage parameters and gain settings of the photomultiplier tube were meticulously adjusted to ensure that the coefficient of variation (CV) for the FITC acceptance channel remained below 5%. Using protoplasts treated with PEG but not transformed with plasmid as the negative control, positive cell populations were screened. Protoplasts containing the target protein were collected through sorting, utilizing a nozzle diameter of 100 μm. Subsequently, a substantial number of positive cells were gathered, and 500 μL of plant cell lysis buffer along with 2.5 mM PMSF reagent were added. The mixture was thoroughly mixed and lysed on ice for 20 min, followed by the collection of the supernatant. Protein quantification was performed using the BCA method, with specific steps executed according to the reagent instructions (Beyotime, P0012S).

### Immunoprecipitation and silver staining analysis

In the immunoprecipitation (IP) experiment, a 50 μL protein sample was combined with 10 μL of 5× SDS protein loading buffer and boiled for 5 min to prepare the input sample. Concurrently, the total protein extracted from protoplasts that did not overexpress PgLEA2-50 served as a blank control. To this protein solution, 15 μL of anti-GFP magnetic bead suspension was added, and the mixture was incubated overnight at 4 °C using a rotary mixing instrument. After incubation, the samples were placed on a magnetic frame for 15 s to allow the beads to separate, and the supernatant was discarded. Subsequently, 600 μL of protein lysate was added, and the magnetic beads were mixed and again placed on the magnetic rack for 15 s to separate and discard the supernatant. This washing step was repeated three times. Finally, 100 μL of SDS‒PAGE loading buffer was added to the magnetic beads, and the mixture was boiled for 5 min. After a 15 s adsorption period, the supernatant was collected. The precipitate was then analyzed using the silver staining method, following the main operational steps as outlined in the manufacturer's instructions (Beyotime, P0017S).

### Protease digestion and mass spectrometry detection

The blocks containing the target bands were washed with ultrapure water and sonicated in a mixture of NH_4_HCO_3_ and acetonitrile for 15 min. This procedure was repeated until complete decolorization was achieved. Subsequently, 100% acetonitrile was added to shake and dehydrate the samples until the colloidal particles turned white. After the liquid was removed, the samples were vacuum-dried for 5 min. Each block was then soaked in an appropriate volume of 10 mM DTT solution until transparent, placed in an oven at 56 °C for 1 h, and subsequently treated with 100 mM iodoacetamide (IAA) solution. This was incubated in the dark for 45 min, followed by two washes with 100 mM NH_4_HCO_3_ and a repetition of the dehydration step. A dilution was prepared with a volume ratio of water:acetonitrile:100 mM NH_4_HCO_3_ = 4:1:5. Trypsin was diluted to 1.2 ng/μL, and an appropriate volume of the enzyme reaction solution was added to the block. The reaction was allowed to proceed for 30 min at 4 °C. After the enzyme solution was absorbed by the block, the residual liquid was discarded, and an excess volume of NH_4_HCO_3_ was added to the system, allowing the block to digest overnight at 37 °C. The gel was then treated with 100 μL of 30% acetonitrile (containing 0.1% trifluoroacetic acid) and sonicated for 15 min. The supernatant was collected and dried in a vacuum at 45 °C. The samples were desalted using a ZipTip C18 micro chromatography column, and the peptides were eluted into a new centrifuge tube and dried. Subsequently, 20 μL of a solution containing 0.1% formic acid and 5% ACN was added. After thorough vortexing, the mixture was centrifuged at 13,000 rpm and 4 °C for 20 min, and the supernatant was collected for mass spectrometry identification. The interacting protein should only be specifically detected in the PgLEA2-50-GFP IP group but not in the IgG control group. PEAKS software was utilized to retrieve and analyze the mass spectrometry data, with the retrieved parameters presented in Table 2. The protein abundance (LFQ intensity) must be significantly higher than the background, with a fold change ≥1.5 and *p* < 0.05 to exclude non-specifically bound proteins.

**Table 2.** Mass spectrometry identification parameter information.

Item	Value
Sequence database search	PEAKS
Protein database	uniprotkb_taxonomy_id_4054_20230424.fasta
Enzyme	Trypsin
Precursor mass tolerance	15 ppm
Fragment mass tolerance	0.05 Da
Dynamic modifications	Oxidation (M); acetylation (N-term)
Static modifications	Carbamidomethylation

### Functional enrichment and pathway mapping of PgLEA2-50 interactome via GO and KEGG databases

The interaction protein gene sequence was uploaded to the eggNOG database (http://eggnog-mapper.embl.de/) to acquire the Gene Ontology (GO) entries and corresponding descriptive information for the target genes.^34^ Utilizing TBtools and the Gene Ontology, the prediction results were filtered and analyzed at level 2.^35^ KEGG annotation information was extracted and organized, and the KEGG metabolic pathway was enriched using the KEGG enrichment analysis module within TBtools. Based on the KEGG annotation background file, the annotation information and gene IDs were iprovided as input, allowing for the analysis of the metabolic pathways of PgLEA2-50 interacting proteins with a significance threshold of *p* < 0.05.

### Comprehensive transcriptomic analysis of protein-coding genes interacting with PgLEA2-50

The transcriptome data of *P. ginseng* under heat, cold, salt, and drought treatments were used to analyze the response of the target genes to major abiotic stress types. These data were obtained from the Korean Ginseng Genome Database (http://ginsengdb.snu.ac.kr/transcriptome.php). Hormone treatments were performed as follows: the seeds of ginseng were cultured at 25 °C under a 16-h light/8-h dark photoperiod. Upon reaching five weeks of age, the seedling leaves were foliar-sprayed with 75 mM 6-BA, 10 mM IAA, 100 mM GA, and 50 mM ABA, respectively. Samples were harvested approximately 5 h post-treatment, with three biological replicates per treatment.

The FastQC procedure was utilized to assess the quality of the raw transcriptome data, while Trimmomatic was employed to eliminate low-quality reads, resulting in clean reads. The genome index of ginseng was built utilizing the Bowtie software (http://bowtie-bio.source forge.net/bowtie2/index.shtml). The filtered sequencing reads were mapped to the relevant loci of the ginseng reference genome via TopHat, after which transcript assembly and splicing were performed using Cufflinks.^36^

Gene expression levels were quantified using the standardized transcripts per million (TPM) value. The expression values of the target gene family from each transcript were extracted from the expression matrix, and a heat map was generated based on the Log_2_ (TPM + 1) values. The counts matrix was imported into DESeq2 (https://bioconductor.org/packages/release/bioc/html/DESeq2.html), enabling the identification of differentially expressed genes based on the criteria of FDR < 0.05 and |Log_2_ Fold-Change|≥1. Subsequently, a volcano plot illustrating the expression of the target gene under different treatments was created.

### Cloning and expression analysis of PgRGA4 in response to GA treatment

In the analysis of transcriptome data, we focused on the transcriptional repression of the core regulatory gene DELLA (PgRGA4, A0A8T9ICW4) within the GA-mediated stress resistance pathway under conditions of high temperature and drought stress (Table 1). Cloning primers for the coding sequence (CDS) region of *PgRGA4* were designed using Primer5 software, and the primer sequence information was listed in Table S2. The gene was cloned utilizing ginseng cDNA as a template and was verified through sequence alignment with the genome.

Five-week-old ginseng seedlings were treated with a 100 mM solution of GA, and RNA was extracted at time points of 0 h, 3 h, 6 h, 12 h, and 24 h. The expression of the *PgRGA4* gene at different periods was detected by quantitative real-time PCR (qRT-PCR). Primers for qRT-PCR detection of *PgRGA4* were designed with β-actin as an internal reference gene (Table S2). The PCR system was 95°C, 30 s; 94°C, 5 s; 60°C, 30 s. The experiment was designed with three biology replications and two technical replications. The experimental data based on Cq values were analyzed by the 2^−∆∆CT^ method and plotted using GraphPad Prism.^37^

### Subcellular localization of PgRGA4

*PgRGA4* was ligated to the pCambia2300-GFP vector using In-Fusion Cloning (pEASY-Basic Seamless Cloning and Assembly Kit), with *SacI* serving as the restriction site. The *PgRGA4-GFP* expression vector was transformed into GV3101 *Agrobacterium* competent cells through heat shock and cultured at 28 °C until the optical density at 600 nm (OD600) reached approximately 0.6. The engineered bacterial solution was then slowly injected into tobacco leaves over a period of about five weeks using a needle-free injection method with a 10 mL syringe. Concurrently, tobacco leaves were transiently transformed with a bacterial solution containing only the empty vector as a control. After 3 d of low-light culture, the expression of the fusion protein was observed using a confocal laser scanning microscope (TCS SP8X, Leica) at a wavelength of 488 nm.

### CO-IP verification of the interaction between PgRGA4 and PgLEA2-50

The pCambia1300 vector, which carries the 3 × FLAG tag, served as the backbone and was linearized using *BamHI* at 37 °C. A seamless cloning primer was designed and synthesized, after which the amplified fragment was ligated to the vector to create a gene expression vector fused with the C-terminus of the PgRGA4 protein. The plasmids *35s-PgRGA4-FLAG* and the previously constructed *35s-PgLEA2-50-GFP* were extracted and quantified to a concentration of 1000 ng/μL. After mixing the two plasmids in equal proportions, they were co-transformed into protoplasts as described in Transient Overexpression of PgLEA2-50 in Ginseng Protoplasts and cultured for 24 h. Total protein was extracted from the sample, then 50 μL was combined with SDS as an input sample, and 15 μL of the pre-treated anti-FLAG immunomagnetic bead suspension was added to the 300 μL sample. The samples were incubated at room temperature for 2 h on a rotary mixer, then separated on a magnetic frame for 10 s, after which the supernatant was discarded. Following this, 300 μL of 1 × TBS solution was added, and the anti-FLAG magnetic beads were re-suspended. Separation was performed again on a magnetic frame for 10 s, and the supernatant was removed. These purification and washing steps were repeated three times. Finally, 100 μL of SDS‒PAGE loading buffer was added to the magnetic beads, which were boiled for 5 min, separated on a magnetic rack for 10 s, and the supernatant was prepared. The total protein of the sample was then separated by 12% polyacrylamide gel electrophoresis, transferred to polyvinylidene difluoride (PVDF) membranes, and blocked in 5% skim milk at 37 °C for 2 h, followed by washing with TBST. The gel images were analyzed after incubation with primary and secondary antibodies.

## Conclusions

The study systematically screened 50 proteins that interact with PgLEA2-50. GO functional annotation and KEGG pathway analysis revealed significant diversity in the biological functions of the interacting proteins, encompassing multiple functional levels, such as substance synthesis and metabolism, stress resistance, and the regulation of antioxidant activity. These proteins play a crucial role in various metabolic pathways and are significantly enriched in essential biological processes, including energy metabolism, oxidative phosphorylation, glycolysis/gluconeogenesis, and photosynthesis. Additionally, they are involved in key signaling pathways, such as plant hormone signal transduction and MAPK signaling pathways. The gene types that interact with the target genes were predominantly concentrated in functional categories, including stress-resistant proteins, transporters, oxidoreductases, antioxidant enzymes, and saponin synthesis. Expression analysis based on transcriptome data further indicates that these gene members exhibit a high degree of responsiveness to abiotic stress. In-depth analysis of the core negative regulator DELLA protein member PgRGA4 within the GA metabolic pathway revealed that this protein is localized in both the nucleus and cytoplasm. Furthermore, its transcription level was inhibited by both GA and stress conditions. The interaction between PgRGA4 and PgLEA2-50 was verified through a CO-IP assay, which not only demonstrated the potential protective effect of PgLEA2-50 on the structure of the DELLA protein but also provided strong experimental evidence for the indirect involvement of PgLEA2-50 in the GA-mediated signaling pathway. These significant findings not only enhance our understanding of the mechanisms underlying ginseng's stress resistance at a theoretical level but also effectively address the issue of production decline faced by the ginseng industry in practical applications, thereby providing important theoretical support and practical technical pathways.

## Supplementary Material

Supplementary material Figure S3.png

Supplementary material Figure S2.jpg

Supplementary material Original images for WB.docx

Supplementary material Table S1.xlsx

Supplementary material Figure S1.jpg

Supplementary material Table S2.xlsx

## Data Availability

The original contributions presented in this study are included in the article/supplementary material. The raw data of the IP-MS analysis are available at https://figshare.com/s/fc241810f62b2b89f5ba (DOI: 10.6084/m9.figshare.31044976). Further inquiries can be directed to the corresponding author(s).

## Abbreviations

LEA: late embryogenesis abundant
GA: gibberellin
GST: glutathione S-transferase
CAT: catalase
LD: linear dichroism
IP-MS: immunoprecipitation-mass spectrometry
CO-IP: co-immunoprecipitation
FDA: fluorescein diacetate
CV: coefficient of variation (CV)
PEG: polyethylene glycol
GO: gene ontology
TPM: transcripts per million
CDS: coding sequence
qRT-PCR: quantitative real-time PCR
PVDF: polyvinylidene difluoride
FSC: forward scatter
SSC: side scatter
PPT: protopanaxatriol
APX: ascorbate peroxidase
ROS: reactive oxygen species
IAA: iodoacetamide
