# Supplementary figures and images for: Deciphering the PgLEA2-50 interactome: implications for abiotic stress responses in Panax ginseng
Source: Plant Signal Behav. 2026 Feb 5;21(1):2624961. doi: 10.1080/15592324.2026.2624961 (PMC12885439; doi:10.1080/15592324.2026.2624961)

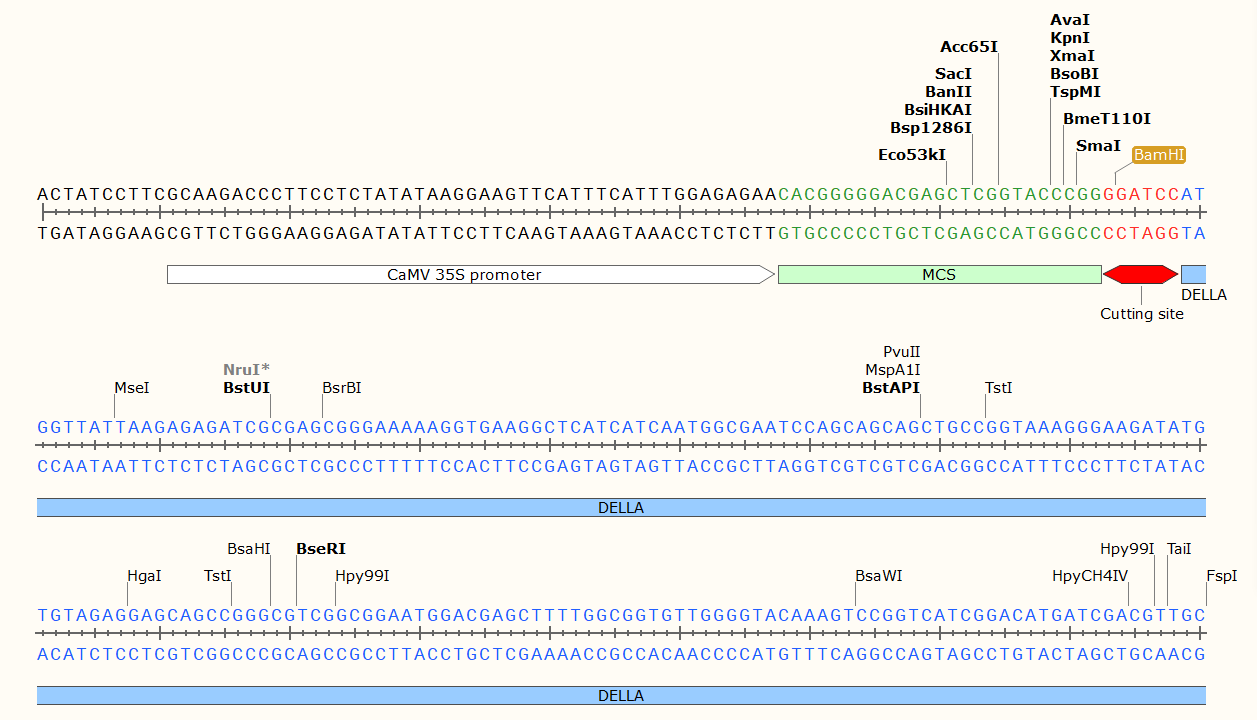

Supplement: Supplementary material — Figure S3.png [file KPSB_A_2624961_SM5390.png]

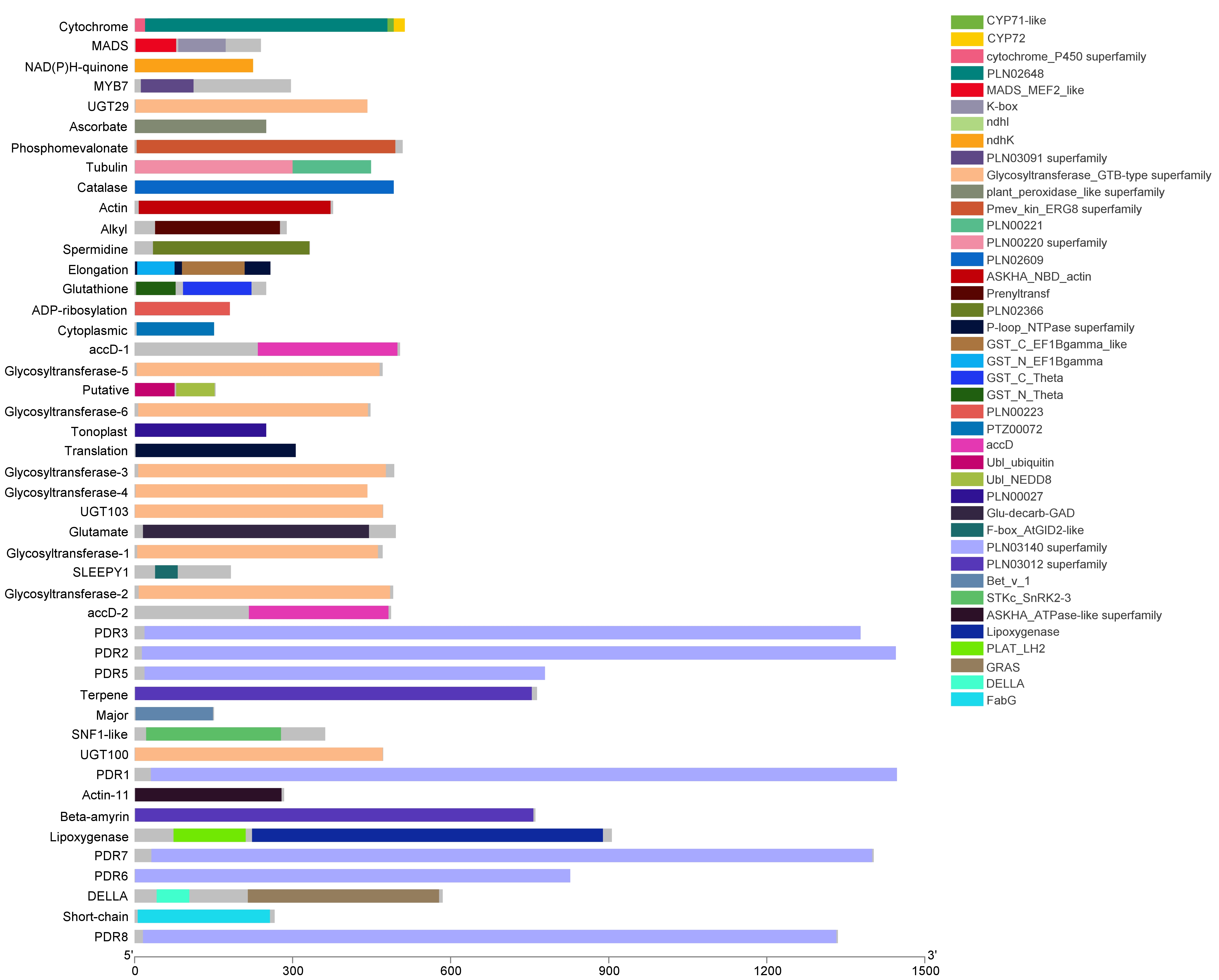

Supplement: Supplementary material — Figure S2.jpg [file KPSB_A_2624961_SM5385.jpg]

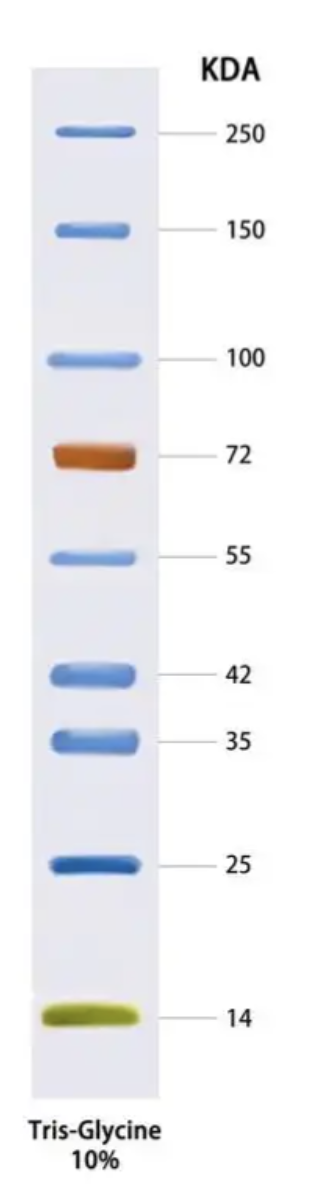


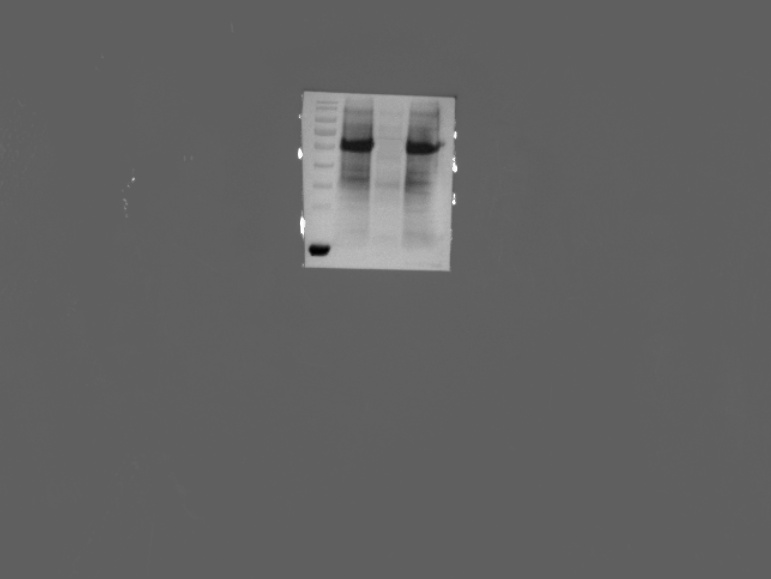


Anti-FLAG-IP


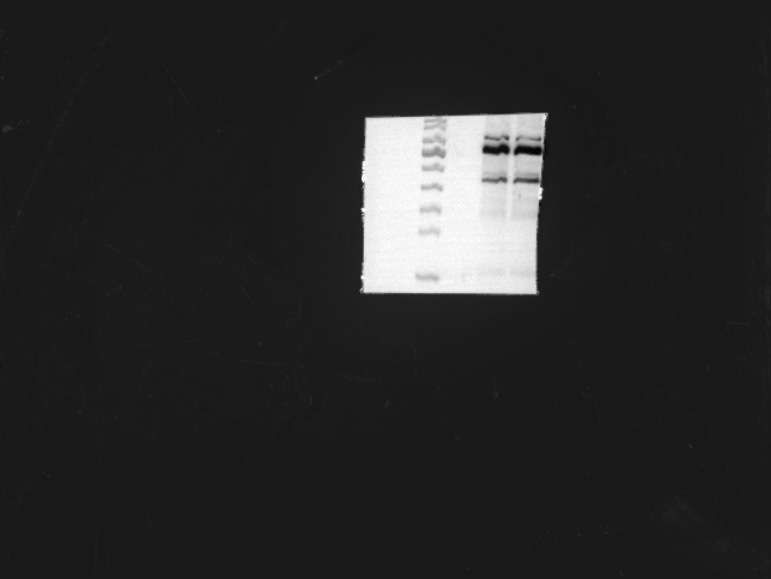


Anti-GFP-Input


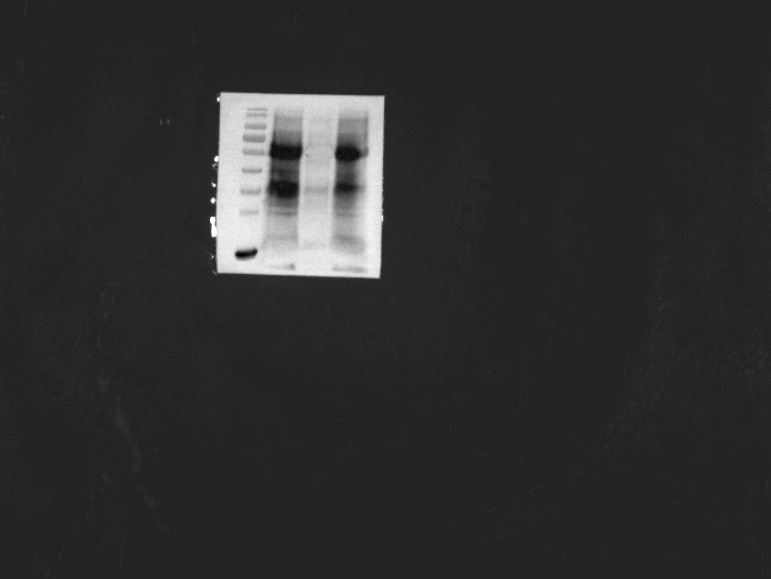


Anti-FLAG-Input


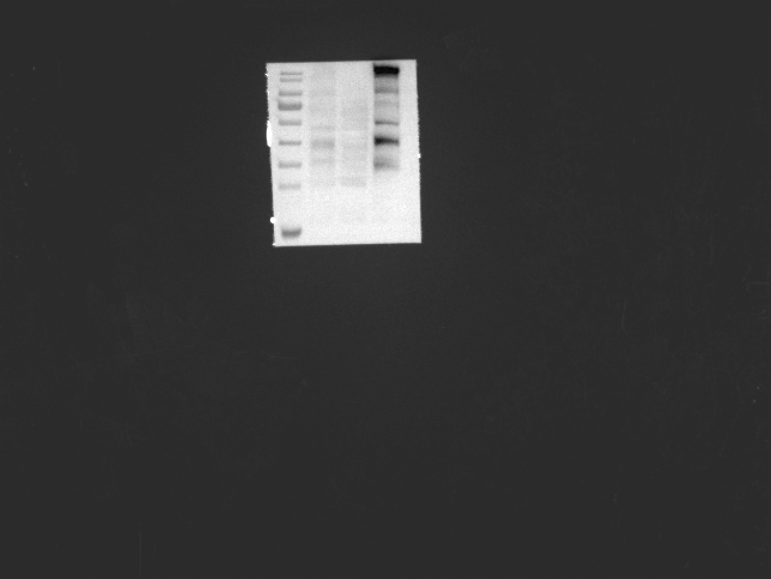


Anti-GFP-IP

Supplement: Supplementary material — Original images for WB.docx [file KPSB_A_2624961_SM5386.docx]

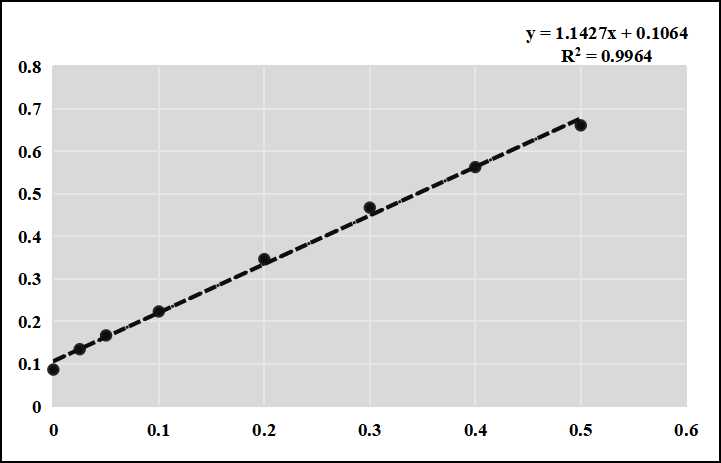

Supplement: Supplementary material — Figure S1.jpg [file KPSB_A_2624961_SM5388.jpg]
